# The SlARF6A‐SlFBA3/SlSGR Module Regulates the Resistance of Tomatoes to *Pseudomonas syringae* pv. *tomato*

**DOI:** 10.1111/mpp.70327

**Published:** 2026-08-21

**Authors:** Tong Pei, Weiman Sun, Tairu Wu, Dalong Li, He Zhang, Xiangyang Xu, Tingting Zhao

**Affiliations:** ^1^ College of Horticulture Northeast Agricultural University Harbin China; ^2^ Key Laboratory of Biology and Genetic Improvement of Horticultural Crops (Northeast Region), Ministry of Agriculture and Rural Affairs Northeast Agricultural University Harbin China

**Keywords:** *Pst* DC3000, *SlARF6A*, *SlFBA3*, *SlSGR*, tomato

## Abstract

Bacterial leaf spot disease is among the most common diseases affecting tomato production. It is caused by *Pseudomonas syringae* pv. *tomato* (Pst) and severely affects tomato plant growth, development, and yield. In this study, we identified a fructose‐1,6‐diphosphate aldolase gene, *SlFBA3*, whose expression was significantly increased in the tomato pathway for resistance to Pst. Overexpression of *SlFBA3* enhanced tomato plant resistance, whereas knockout of *SlFBA3* reduced the resistance of tomato plants. Further analysis revealed that SlSGR is an interacting protein of SlFBA3. Functional analysis showed that overexpression of *SlSGR* inhibited the resistance of tomato to Pst, whereas knockout of *SlSGR* increased resistance. Mechanistically, upon flg22 and csp22 elicitation, *SlFBA3* can enhance the early immune signalling pathway, while *SlSGR* inhibits this response. Notably, we found that SlFBA3 can partially antagonize the function of SlSGR, thereby weakening the negative regulatory role of *SlSGR* in the disease response. In addition, we discovered that the positive regulatory factor *SlARF6A* of this disease‐resistant response can target and regulate the transcriptional activation activity of *SlFBA3*. In conclusion, we revealed that the SlARF6A–SlFBA3/SlSGR module plays an important role in the resistance response of tomato to Pst, providing a new research direction for disease resistance breeding in tomato.

## Graphical abstract

The SlARF6A‐SlFBA3/SlSGR module plays an important role in the resistance response of tomato to *Pseudomonas syringae* pv. *tomato* (Pst) DC3000. The transcription factor *SlFBA3*, which is targeted and activated by SlARF6A, antagonizes the negative regulatory factor *SlSGR*, thereby enhancing the resistance of tomato to Pst DC3000.

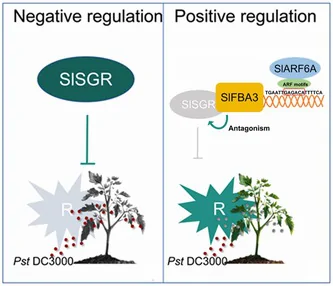

## 1 Introduction

Tomato ( *Solanum lycopersicum* ), one of the most economically important vegetable crops worldwide, holds a significant position in the agricultural industrial structure (Yu et al. 2025). However, tomato production is severely threatened by various bacterial diseases, among which bacterial speck caused by *Pseudomonas syringae* pv. *tomato* (Pst) represents one of the most destructive and widespread diseases affecting tomato cultivation globally (Xin and He 2013; Basim et al. 2004). This pathogen primarily infects aboveground tissues, including leaves, stems and fruits, leading to tissue necrosis, reduced photosynthetic capacity, and consequently severe impairment of plant growth and development, resulting in substantial losses in both yield and fruit quality. Pst strain DC3000 serves as a ‘model organism’ for studying plant–pathogen interactions and is widely used to investigate the mechanisms of disease occurrence and plant immune pathways (Xin and He 2013). Despite considerable progress made using this model system, the intricate regulatory networks governing tomato defence against Pst remain to be fully elucidated.

Plants deploy a two‐layered innate immune system to defend against Pst. The first layer is pattern‐triggered immunity (PTI), which is activated when plasma membrane‐localized pattern recognition receptors (PRRs) perceive conserved pathogen‐associated molecular patterns (PAMPs) or damage‐associated molecular patterns (DAMPs), thereby initiating basal defence responses (Boller and Felix 2009; Schwessinger and Ronald 2012). To counteract PTI, plants have evolved a second layer, namely, effector‐triggered immunity (ETI), which relies on intracellular nucleotide‐binding leucine‐rich repeat (NLR) receptors to recognize specific effectors (Jones and Dangl 2006). A well‐characterized ETI module in tomato is the *Pto* gene, which encodes a protein kinase that can trigger strong resistance by recognizing the Pst effector protein AvrPto/AvrPtoB (Mathieu et al. 2014). Notably, plants lacking the *Pto* gene are unable to recognize AvrPto/AvrPtoB through the Pto/Prf pathway, highlighting the specificity of the ETI pathway (Oh and Martin 2011). Activation of PTI and ETI triggers a series of downstream defence responses, including transcriptional reprogramming, production of reactive oxygen species (ROS), mitogen‐activated protein kinase (MAPK) cascade reactions and phytohormone signalling, ultimately leading to enhanced disease resistance.

Fructose‐1,6‐bisphosphate aldolase (FBA) is a key enzyme in glycolysis and the Calvin cycle. Members of the FBA family not only participate in basic metabolic processes but also respond to various abiotic stresses, including drought stress, salt stress and low temperatures (Zhang et al. 2025). For example, *MsFBA6* participates in the cold stress response through the calcium signalling pathway mediated by the calcium‐binding protein MsCML10 and cooperates with MsGSTU8 to regulate the homeostasis of ROS and sugar accumulation‐mediated osmotic regulation (Yu et al. 2022). In *Brassica campestris* , *BcFBA8* can be targeted and activated by BcNAC55.2, thereby reducing the accumulation of ROS in the plant and enhancing its antioxidant capacity, and ultimately improving the plant's tolerance to stress conditions such as drought, salt and high temperature (Wang et al. 2025). In tomato, a genome‐wide analysis revealed that there are eight *SlFBA* genes in tomatoes, which are further divided into two subgroups based on phylogenetic relationships, gene structure, and conserved motifs (Cai et al. 2016). Expression profiling analysis indicated that all *SlFBA* members are involved in the response to low and high temperature stresses (Cai et al. 2016, 2018, 2022). However, few studies have investigated its regulatory role in biotic stress, especially in disease caused by Pst. Therefore, in this study, we aimed to explore the function and molecular mechanism of *SlFBA3* in the defence response pathway of tomato against Pst.

STAY‐GREEN (SGR) family proteins have been identified as key factors involved in chlorophyll degradation and leaf senescence (Sakuraba et al. 2012). In recent years, members of this family have been shown to play important roles in plant immune regulation. For example, in *Arabidopsis thaliana* , *AtSGR* mutants exhibit stronger resistance to Pst DC3000, and the symptoms of leaf chlorosis are significantly alleviated after pathogen infection (Mur et al. 2010; Mecey et al. 2011). In tomato, *SlSGR* mutants exhibit significantly enhanced resistance to *Botrytis cinerea* (Gianoglio et al. 2022). In cucumber, natural variation or gene‐editing mutants of the *CsSGR* gene exhibit broad‐spectrum resistance to different pathogens, and this resistance involves both passive defence (such as delaying chlorophyll degradation and senescence) and active defence (such as the activation of the salicylic acid [SA] and jasmonic acid [JA] signalling pathways) (Tan et al. 2026). In rice, a natural mutation of *OsSGR* enhances resistance to *Rhizoctonia solani* by inhibiting the expression of cytokinin degradation genes (Xie et al. 2025). Despite these advances, the molecular mechanisms and regulatory modules through which SGR proteins modulate disease resistance remain largely unexplored, especially in tomato.

Auxin response factors (ARFs) are transcription factors that activate or inhibit the expression of target genes by binding to auxin response elements in the promoter regions of these genes. Upon pathogen infection, certain ARFs have been shown to regulate defence‐related gene expression (Qin et al. 2020). For example, ARFs have been shown to activate transcription factors such as OsWRKY13 to enhance antiviral immunity (Qin et al. 2020). In rice, *OsARF17* has been reported to play a crucial role in antiviral defence, participating in the response to rice stripe mosaic virus by regulating the expression of multiple defence‐related genes (WRKY, *OsAHT2* and *OsDR8*) (Ma et al. 2023). These findings demonstrate that ARFs can positively regulate plant defence by directly activating downstream genes involved in various defence processes.

In this study, we identified a regulatory module SlARF6A–SlFBA3/SlSGR that plays a crucial role in tomato resistance to Pst. We demonstrate that *SlFBA3*, transcriptionally activated by SlARF6A, promotes resistance by antagonizing the negative regulator SlSGR. These findings reveal a novel mechanism of fine‐tuning immune responses in tomato and provide a potential target for breeding disease‐resistant cultivars.

## 2 Results

### 2.1 Expression Pattern and Subcellular Localization of *SlFBA3*

**FIGURE 1:**
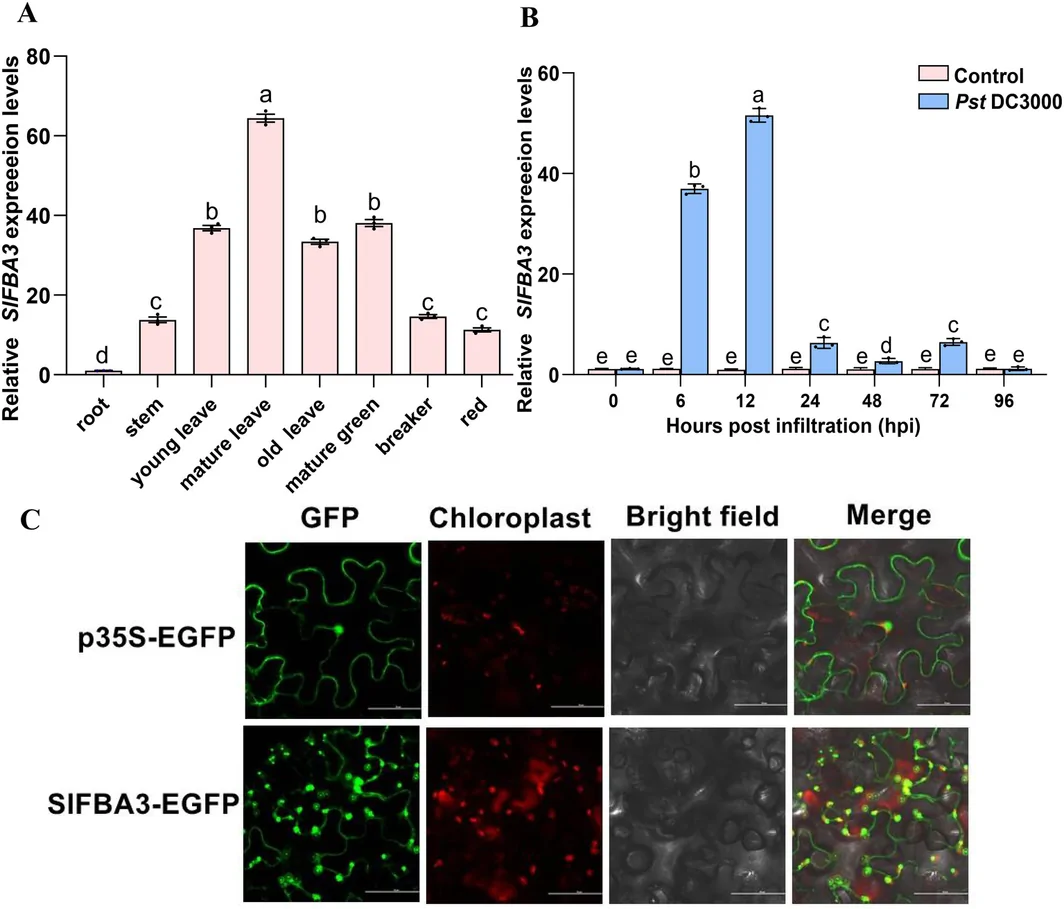
Expression pattern and subcellular localization of *SlFBA3*. (A) Tissue‐specific expression of *SlFBA3*. Young leaves, leaves at the top of the plant that have just grown; mature leaves, the fifth leaf fully unfolded from the top of the plants; old leaves, the yellowing leaves at the bottom of the plants. (B) *SlFBA3* expression at different time points after *Pseudomonas syringae* pv. *tomato* (Pst) DC3000 was sprayed. (C) Subcellular localization of SlFBA3 in *Nicotiana benthamiana* leaf cells. Bar = 50 μm. One‐way ANOVA with Tukey's HSD; different letters indicate significant differences (*p* < 0.05). The error bars denote SDs (*n* = 3).

To investigate the expression pattern of *SlFBA3*, we examined its expression levels in various tomato tissues. The expression level of *SlFBA3* was higher in leaves than in fruits, stems, and roots. The expression levels in young and old leaves were lower than that in mature leaves (Figure 1A). To determine whether *SlFBA3* is involved in tomato's resistance to Pst DC3000, we measured the expression level of *SlFBA3* after the tomatoes were inoculated with Pst DC3000. We found that the expression level of *SlFBA3* significantly increased at 6 and 12 h post‐infiltration (hpi), with rapid upregulation of more than 30‐ and 50‐fold, respectively. At 24 hpi, the expression level rapidly decreased to near the level before inoculation (Figure 1B). Subcellular localization provides crucial clues for understanding protein function, as it determines the potential interacting partners and the appropriate yeast two‐hybrid (Y2H) system to test these. To gain insight into the function of SlFBA3 protein, we examined its subcellular distribution. The results showed that SlFBA3‐EGFP fusion protein was expressed in the chloroplasts of *Nicotiana benthamiana* cells, and a small amount of GFP signal was detected in the cell membrane (Figure 1C).

### 2.2 *SlFBA3* Positively Regulates the Resistance of Tomatoes to Pst DC3000

**FIGURE 2:**
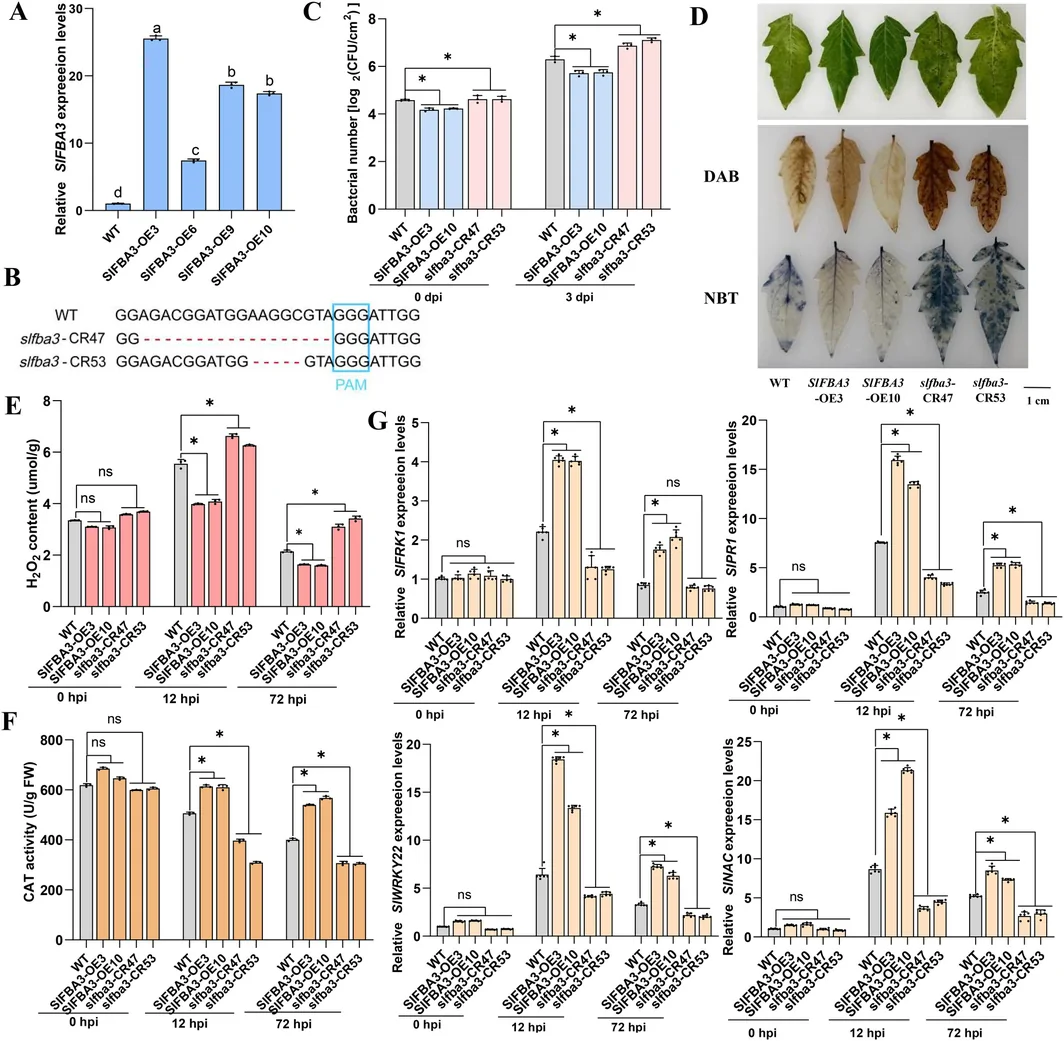
*SlFBA3* positively regulates the resistance of tomatoes to *Pseudomonas syringae* pv. *tomato* DC3000. (A) The expression levels of *SlFBA3* in overexpression (OE) lines and wild type (WT). One‐way ANOVA with Tukey's HSD; different letters indicate significant differences (*p* < 0.05). The error bars denote SDs (*n* = 3). (B) The types of mutations in two CRISPR/Cas9‐edited (CR) lines. The red dashed lines represent nucleotide or amino acid deletions. Mutants CR47 and CR53 had an 18 bp or 5 bp deletion, respectively. (C) Bacterial counts on leaves of the WT, OE and CR lines were measured at 0 and 3 days post‐inoculation (dpi). (D) Phenotypes, 3,3′‐diaminobenzidine (DAB) staining and nitroblue tetrazolium (NBT) staining of the WT, OE and CR lines at 3 dpi. (E) H_2_O_2_ content of the WT, OE and CR lines at 0, 12 and 72 hpi. (F) Catalase (CAT) activity of the WT, OE and CR lines at 0, 12 and 72 h post‐inoculation (hpi). (G) The expression levels of the disease‐resistance genes *SlFRK1*, *SlPR1*, *SlWRKY22* and *SlNAC* in the WT, OE and CR lines at 0, 12 and 72 hpi. Asterisks indicate a significant difference (ns, not significant; *p < 0.05, **p < 0.01, Student's *t*‐test). The error bars denote SDs (*n* = 6).

To clarify the function of *SlFBA3* in tomato resistance to Pst DC3000, we constructed *SlFBA3* overexpression lines (*SlFBA3*‐OE3 and *SlFBA3*‐OE10) and CRISPR/Cas9‐edited mutant lines (*slfba3*‐CR47 and *slfba3*‐CR53) (Figure 2A,B, Figure S1). In this study, we found that after inoculation with Pst DC3000, compared with those of the wild type (WT), the leaves of the *SlFBA3* CR mutants presented more lesions and more severe necrosis, a significantly greater number of bacteria on the leaves, increased accumulation of H_2_O_2_ and O^2−^, as indicated by 3,3′‐diaminobenzidine (DAB) and nitroblue tetrazolium (NBT) staining, respectively (Figure 2C,D). However, compared with the WT, the *SlFBA3* overexpression lines presented fewer lesions and less necrosis, fewer bacteria in the leaves, and less accumulation of H_2_O_2_ and O_2_^−^ (Figure 2C,D). ROS play a crucial role as a signalling module in pathogen–plant interactions (Heller and Tudzynski 2011). At 0 hpi, there was no significant difference in H_2_O_2_ content and catalase (CAT) activity observed between WT and *SlFBA3* overexpression lines. At 12 hpi, the H_2_O_2_ content in the *SlFBA3* overexpression lines was significantly lower than that of the WT, and the CAT activity was significantly higher than that of the WT. However, the *SlFBA3* CR mutants were the opposite (Figure 2E,F). In addition, the expression levels of the disease resistance genes *SlFRK1*, *SlPR1*, *SlWRKY22* and *SlNAC* in the *SlFBA3* overexpression lines were significantly higher than those in the WT, while the expression levels of disease resistance genes in the *SlFBA3* CR mutants were significantly lower than those in the WT (Figure 2G). These results indicated that *SlFBA3* is a positive regulatory factor for the resistance response of tomato to Pst DC3000.

### 2.3 SlFBA3 Interacts With SlSGR

**FIGURE 3:**
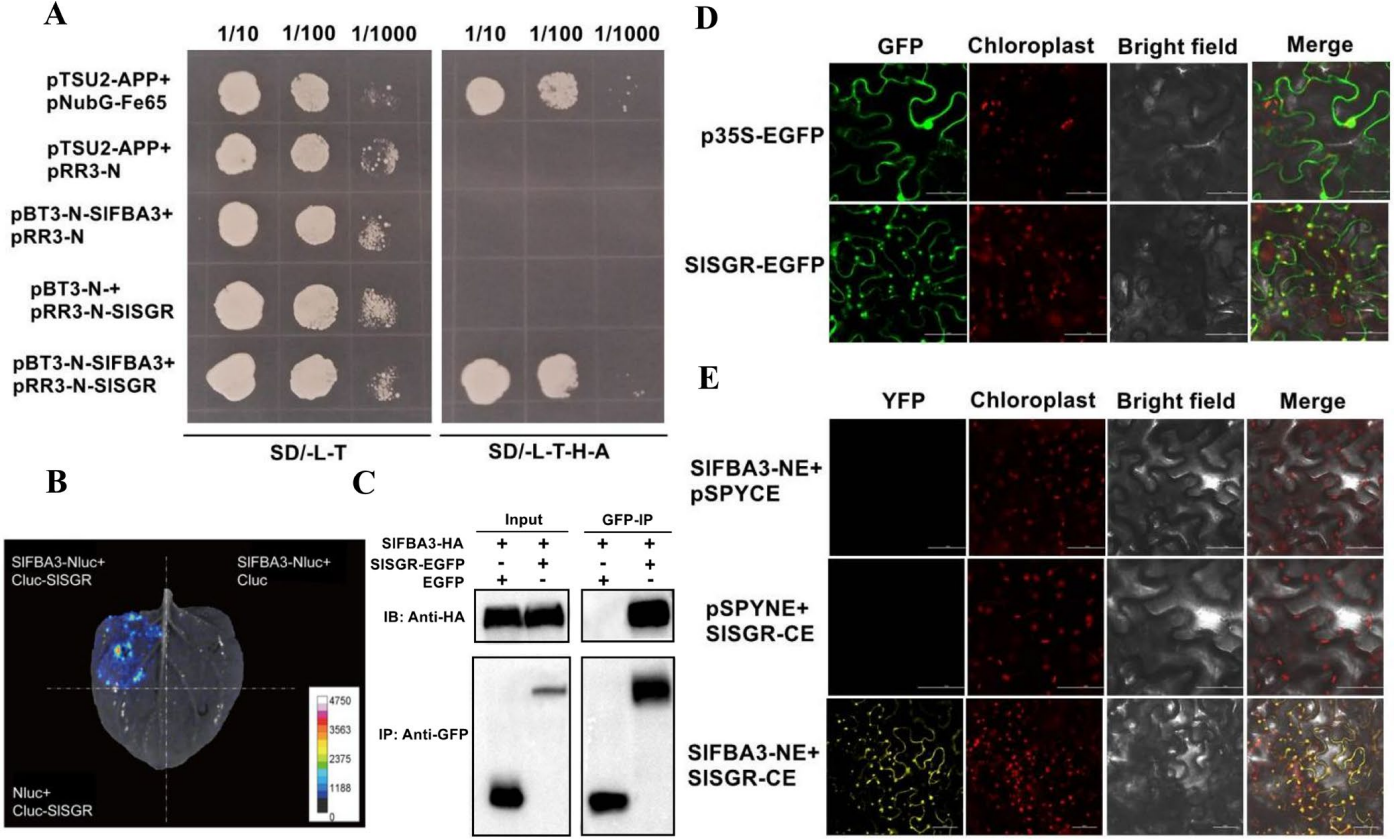
SlFBA3 interacts with SlSGR. (A) Yeast two‐hybrid assay verified the interaction between SlFBA3 and SlSGR, pTSU2‐APP+pNubG‐Fe65 was used as positive control, pTSU2‐APP+pRR3‐N, pBT3‐N‐*SlFBA3*+pRR3‐N and pBT3‐N‐+pRR3‐N‐*SlSGR* were used as negative controls. (B) Luciferase complementation imaging (LCI) assay verified the interaction between SlFBA3 and SlSGR. (C) Co‐immunoprecipitation (Co‐IP) assay was used to validate the interaction between SlFBA3 and SlSGR. (D) Subcellular localization of SlSGR in *Nicotiana benthamiana* leaf cells. (E) Bimolecular fluorescence complementation (BiFC) assay verified the interaction between SlFBA3 and SlSGR in *N. benthamiana*. *SlFBA3*‐NE+pSPYCE and pSPYNE+*SlSGR*‐CE were used as negative controls. Bar = 50 μm.

To explore the regulatory mechanism of *SlFBA3* in the disease resistance pathway, we screened a cDNA library. The Y2H results indicated that pBT3‐N‐*SlFBA3*+pPR3‐N‐*SlSGR* were able to grow on SD/−Leu−His−Trp−Ade selective medium, suggesting that SlFBA3 interacted with SlSGR (Figure S2, Figure 3A). In a luciferase complementation imaging (LCI) experiment, after *N. benthamiana* leaves were sprayed with D‐luciferin solution, luciferase signals were observed in leaves coexpressing SlFBA3‐Nluc and SlSGR*‐*Cluc (Figure 3B). Co‐immunoprecipitation (Co‐IP) experiments also confirmed that SlSGR could be coprecipitated with SlFBA3 (Figure 3C). Subcellular localization of the SlSGR‐EGFP fusion protein localized to the chloroplasts and cell membranes of *N. benthamiana* (Figure 3D). In a bimolecular fluorescence complementation (BiFC) experiment, YFP fluorescence signals were produced in the chloroplasts and cell membranes of *N. benthamiana* leaves expressing SlFBA3‐NE and SlSGR‐CE (Figure 3E). These results indicate that SlFBA3 and SlSGR can interact with each other both in vivo and in vitro.

### 2.4 *SlSGR* Negatively Regulates the Resistance of Tomatoes to Pst DC3000

**FIGURE 4:**
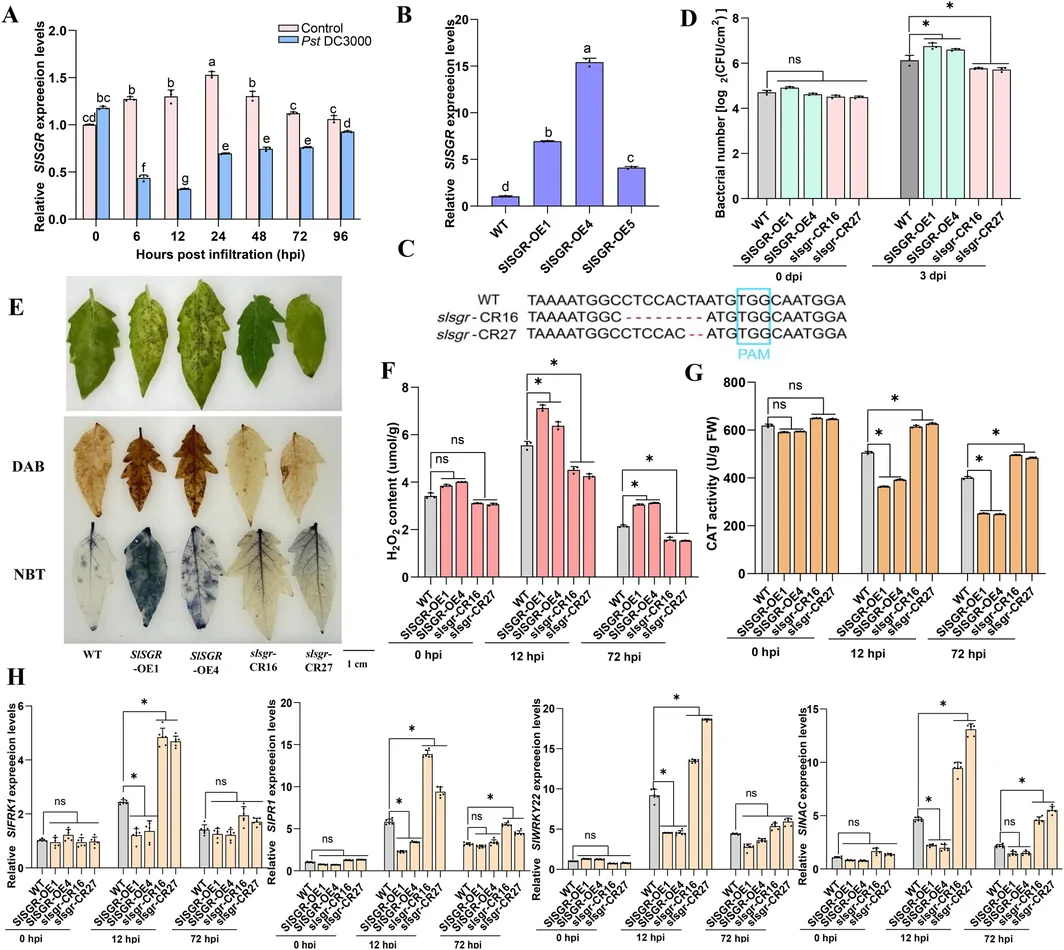
*SlSGR* negatively regulates the resistance of tomatoes to *Pseudomonas syringae* pv. *tomato* DC3000. (A) *SlSGR* expression at different time points after the pathogen was sprayed. One‐way ANOVA with Tukey's HSD; different letters indicate significant differences (*p* < 0.05). The error bars denote SDs (*n* = 3). (B) The relative expression levels of *SlSGR* in overexpression lines (OE) and wild type (WT). (C) The types of mutations in two CRISPR/Cas9‐edited (CR) lines. The red dashed lines represent nucleotide or amino acid deletions. Mutants CR16 and CR27 had an 8 bp or 2 bp deletion, respectively. (D) Bacterial counts on leaves of the WT, OE and CR lines were measured at 0 and 3 days post‐inoculation (dpi). (E) Phenotypes, 3,3′‐diaminobenzidine (DAB) staining and nitroblue tetrazolium (NBT) staining of the WT, OE, and CR lines at 3 dpi. (F) H_2_O_2_ content of the WT, OE and CR lines at 0, 12 and 72 h post‐inoculation (hpi). (G) Catalase (CAT) activity of the WT, OE and CR lines at 0, 12 and 72 hpi. (H) The expression levels of the disease‐resistance genes *SlFRK1*, *SlPR1*, *SlWRKY22* and *SlNAC* in the WT, OE and CR lines at 0, 12 and 72 hpi. Asterisks indicate a significant difference (ns, not significant; *p < 0.05, **p < 0.01, Student's *t‐*test). The error bars denote SDs (*n* = 6).

We examined the expression pattern of *SlSGR* and found that its expression level significantly decreased after Pst DC3000 infection (Figure 4A). To clarify the function of the *SlSGR* gene in the disease resistance response, we generated *SlSGR*‐overexpression (*SlSGR‐*OE1, *SlSGR‐*OE4 and *SlSGR‐*OE5) and *SlSGR*‐CRISPR/Cas9‐edited lines (*slsgr*‐CR16 and *slsgr*‐CR27) (Figure 4B,C, Figure S3). T_2_ plants with relatively high expression levels were selected from the lines, namely, *SlSGR*‐OE1 and *SlSGR*‐OE4, for subsequent studies. After Pst DC3000 inoculation, compared with those of the WT plants, the leaves of the *SlSGR* overexpression lines presented more lesions and more severe necrosis, significantly increased numbers of bacteria on the leaves, increased accumulation of H_2_O_2_ and O_2_^−^ (Figure 4D,E). However, compared with the WT, the *SlSGR* CR mutants presented fewer leaf lesions and necrosis, fewer bacteria on the leaves, and lower accumulation of H_2_O_2_ and O_2_^−^ (Figure 4D,E). The ROS results indicated that the antioxidant capacity of the *SlSGR* overexpression lines was lower than that of the WT, while the antioxidant capacity of the *SlSGR* CR mutants was higher than that of the WT (Figure 4F,G). The expression level of disease resistance genes in *SlSGR* overexpression lines was significantly lower than that of the WT, while they were higher in *SlSGR* CR mutants than in the WT (Figure 4H). These results indicated that *SlSGR* negatively regulates the resistance of tomato to Pst DC3000.

To explore whether the *SlFBA3/SlSGR* module is involved in broad‐spectrum resistance, we tested the resistance of *SlFBA3* and *SlSGR* transgenic lines to *Xanthomonas euvesicatoria* . In contrast to the results observed with Pst DC3000, there was no significant difference in disease resistance of the transgenic lines compared to the WT (Figure S4). This indicates that the resistance response mediated by the *SlFBA3/SlSGR* module does not participate in the broad‐spectrum resistance of plants.

### 2.5 *SlFBA3* and *SlSGR* Regulate Immune Response in Tomato

**FIGURE 5:**
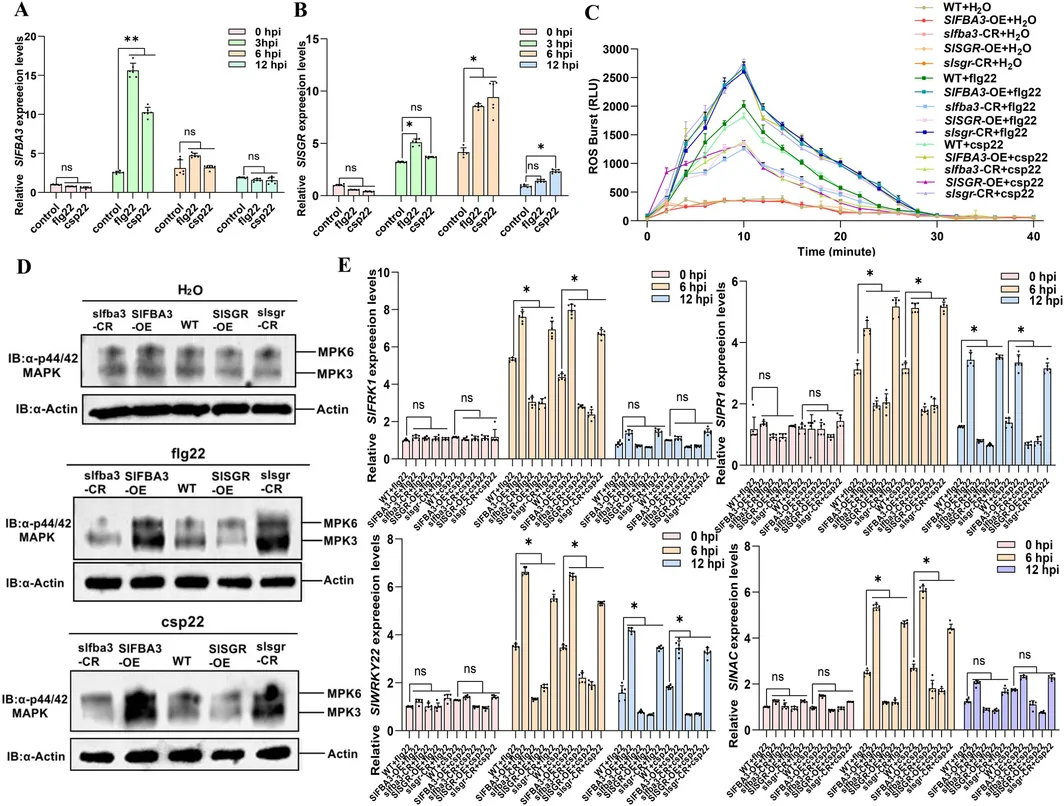
*SlFBA3* and *SlSGR* regulate immune response in tomato. (A) The relative expression levels of *SlFBA3* after treatment with water, flg22 (1 μM) or csp22 (1 μM) for 0, 3, 6 and 12 h. (B) The relative expression levels of *SlSGR* after treatment with water, flg22 (1 μM) or csp22 (1 μM) for 0, 3, 6 and 12 h. (C) Reactive oxygen species (ROS) production induced by flg22 or csp22 in wild‐type (WT), overexpression lines (*SlFBA3*‐OE, *SlSGR*‐OE) and CRISPR/Cas9‐mutated (*SlFBA3*‐CR, *SlSGR*‐CR) lines. ROS production was detected as relative luminescence units (RLU). (D) Immunoblotting of phosphorylated MAPK using anti‐p42/44 MAPK antibody. WT, *SlFBA3*‐OE, *SlFBA3*‐CR, *SlSGR*‐OE and *SlSGR*‐CR lines were treated with water, 1 μM flg22 or 1 μM csp22 (15 min). Anti‐Actin antibody was used as a loading control. (E) The expression levels of *SlFRK1*, *SlPR1*, *SlWRKY22* and *SlNAC* genes in WT, *SlFBA3*‐OE, *SlFBA3*‐CR, *SlSGR*‐OE and *SlSGR*‐CR lines at 0, 6 and 12 h post‐inoculation (hpi). Tomato plant leaves were treated with 1 μM flg22 or 1 μM csp22. Asterisks indicate a significant difference (ns, not significant; *p < 0.05, **p < 0.01, Student's *t*‐test). The error bars denote SDs (*n* = 6).

To investigate the regulatory roles of *SlFBA3* and *SlSGR* in the immune response, we measured the expression levels of *SlFBA*3 and *SlSGR* genes after treatment with flg22 (1 μM) and csp22 (1 μM). The transcript levels of both genes remained relatively constant across all time points. In contrast, upon flg22 and csp22 treatment, the expression of *SlFBA3* was rapidly and strongly induced, reaching a peak at 3 hpi, and then gradually decreasing. The expression of *SlSGR* was also significantly upregulated by flg22 and csp22 treatments, but delayed slightly, peaking at 6 hpi (Figure 5A,B). The ROS burst kinetics (0–40 min) revealed that after treatment with flg22 or csp22, the *SlFBA3* overexpression lines and *SlSGR* mutants exhibited significantly stronger and more rapid ROS bursts compared to the WT, whereas the ROS bursts in *SlFBA3* mutants and *SlSGR* overexpression lines were markedly lower than those of the WT (Figure 5C). These results indicate that *SlFBA3* promotes the early ROS signalling pathway triggered by the immune response, while *SlSGR* inhibits the ROS signalling pathway. In addition, in the *SlFBA3* overexpression lines and *SlSGR* mutants treated with flg22 or csp22, the phosphorylation levels of MPK6/MPK3 (detected by phosphorylated p44/42 antibody) were significantly higher than those of the WT, while the *SlFBA3* mutants and the *SlSGR* overexpression lines weakened the activation of MAPK (Figure 5D). Next, we examined the expression of four immune‐related marker genes, *SlFRK1*, *SlWRKY22*, *SlPR1* and *SlNAC*. After treatment with flg22 and csp22, the transcriptional levels of these four marker genes in the *SlFBA3* overexpression lines and *SlSGR* mutants were significantly higher than those of the WT, while the opposite was observed in the *SlFBA3* mutants and *SlSGR* overexpression lines (Figure 5E). These data indicate that *SlFBA3* plays a positive regulatory role in the immune response of tomato, while *SlSGR* exerts a negative regulatory effect.

### 2.6 SlFBA3 Antagonizes the Function of SlSGR

**FIGURE 6:**
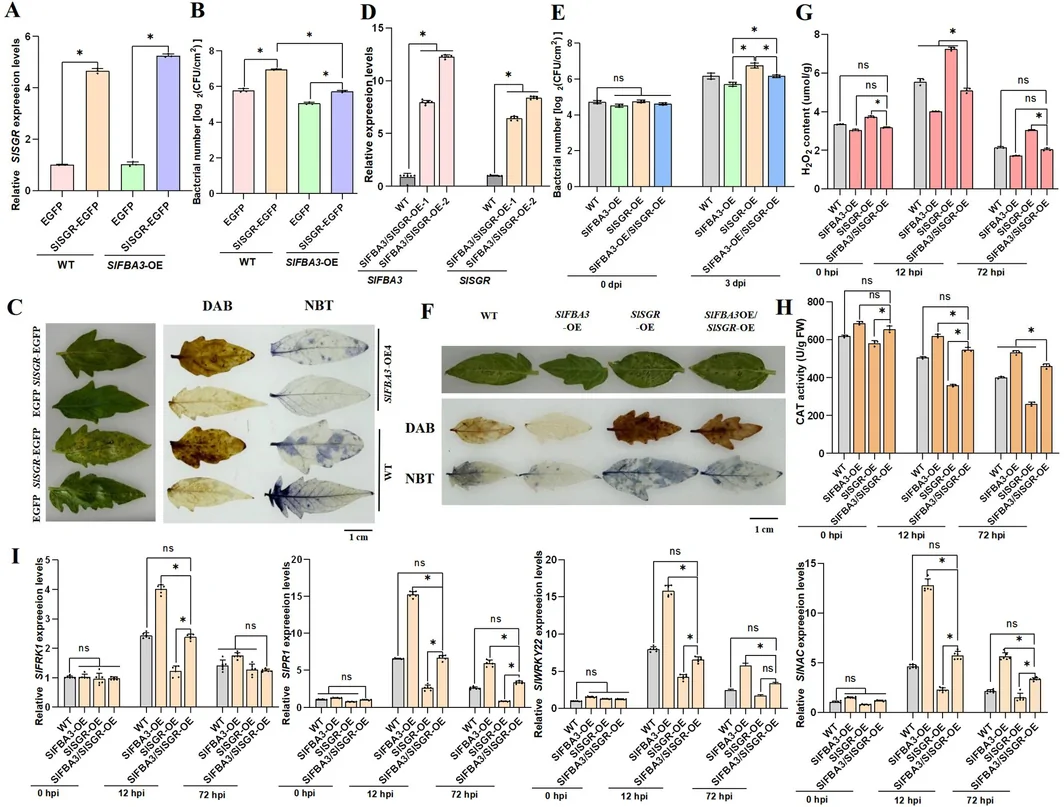
*SlFBA3* antagonizes the function of *SlSGR*. (A) The relative expression levels of *SlSGR* in wild‐type (WT) and *SlFBA3*‐OE (overexpression) lines. (B) The bacterial counts of transient expression of EGFP and *SlSGR*‐EGFP in WT and *SlFBA3*‐OE lines at 3 days post‐inoculation (dpi). (C) The phenotypes, 3,3′‐diaminobenzidine (DAB) staining and nitroblue tetrazolium (NBT) staining of transient expression of EGFP and *SlSGR*‐EGFP in WT and *SlFBA3*‐OE lines at 3 dpi. (D) The relative expression levels of *SlFBA3* and *SlSGR* genes in WT and *SlSGR*/*SlFBA3*‐OE lines. (E) The bacterial counts of the WT, *SlFBA3*‐OE, *SlSGR*‐OE and *SlFBA3*‐OE/*SlSGR*‐OE lines at 3 dpi. (F) The phenotypes, DAB staining and NBT staining of the WT, *SlFBA3*‐OE, *SlSGR*‐OE and *SlFBA3*‐OE/*SlSGR*‐OE at 3 dpi. (G) H_2_O_2_ content of the WT, *SlFBA3*‐OE, *SlSGR*‐OE and *SlFBA3*‐OE/*SlSGR*‐OE lines at 0, 12 and 72 h post‐inoculation (hpi). (H) Catalase (CAT) activity of the WT, *SlFBA3*‐OE, *SlSGR*‐OE and *SlFBA3*‐OE/*SlSGR*‐OE lines at 0, 12 and 72 hpi. (I) The expression levels of the disease‐resistance genes *SlFRK1*, *SlPR1*, *SlWRKY22* and *SlNAC* in the WT, *SlFBA3*‐OE, *SlSGR*‐OE and *SlFBA3*‐OE/*SlSGR*‐OE at 0, 12 and 72 hpi. Asterisks indicate a significant difference (ns, not significant; *p < 0.05, **p < 0.01, Student's *t*‐test). The error bars denote SDs (*n* = 6).

To investigate the role of the SlFBA3/SlSGR interaction module in the resistance to Pst DC3000, we transiently expressed EGFP and *SlSGR*‐EGFP in WT and *SlFBA3*‐OE plants. At 72 hpi, *SlSGR* expression was confirmed (Figure 6A). Subsequently, these plants were inoculated with Pst DC3000. Compared with the transient expression of *SlSGR*‐EGFP in WT plants, the transient expression of *SlSGR*‐EGFP in *SlFBA3*‐OE plants reduced the degree of lesions and necrosis and decreased bacterial numbers on the leaves (Figure 6B,C). Moreover, the accumulation levels of H_2_O_2_ and O_2_^−^ were decreased (Figure 6C). This indicates that SlFBA3 can partially weaken the negative regulatory effect of SlSGR.

To test the antagonistic effect of the functions of SlFBA3 and SlSGR at the genetic level, we constructed *SlFBA3*‐OE/*SlSGR*‐OE double overexpression lines by crossing *SlFBA*3‐OE with *SlSGR*‐OE (Figure 6D). Western blot confirmed the stable accumulation of SlFBA3 and SlSGR in the double overexpression lines (Figure S5). Disease resistance analysis showed that the resistance of the *SlFBA3*‐OE/*SlSGR*‐OE double overexpression lines was between that of *SlFBA3*‐OE and *SlSGR*‐OE, which was consistent with the above results (Figure 6E,F,I). Compared with the single *SlSGR*‐OE lines, the *SlFBA3*‐OE/*SlSGR*‐OE double overexpression lines showed a significant increase in CAT activity and a decrease in H_2_O_2_ accumulation (Figure 6G,H). These results indicate that the positive regulatory factor SlFBA3 could partially antagonize the function of the negative regulatory factor SlSGR, thereby affecting the disease resistance of tomato to Pst DC3000.

### 2.7 SlARF6A Binds to the Promoter of *SlFBA*3 and Positively Regulates Tomato Resistance to Pst DC3000

**FIGURE 7:**
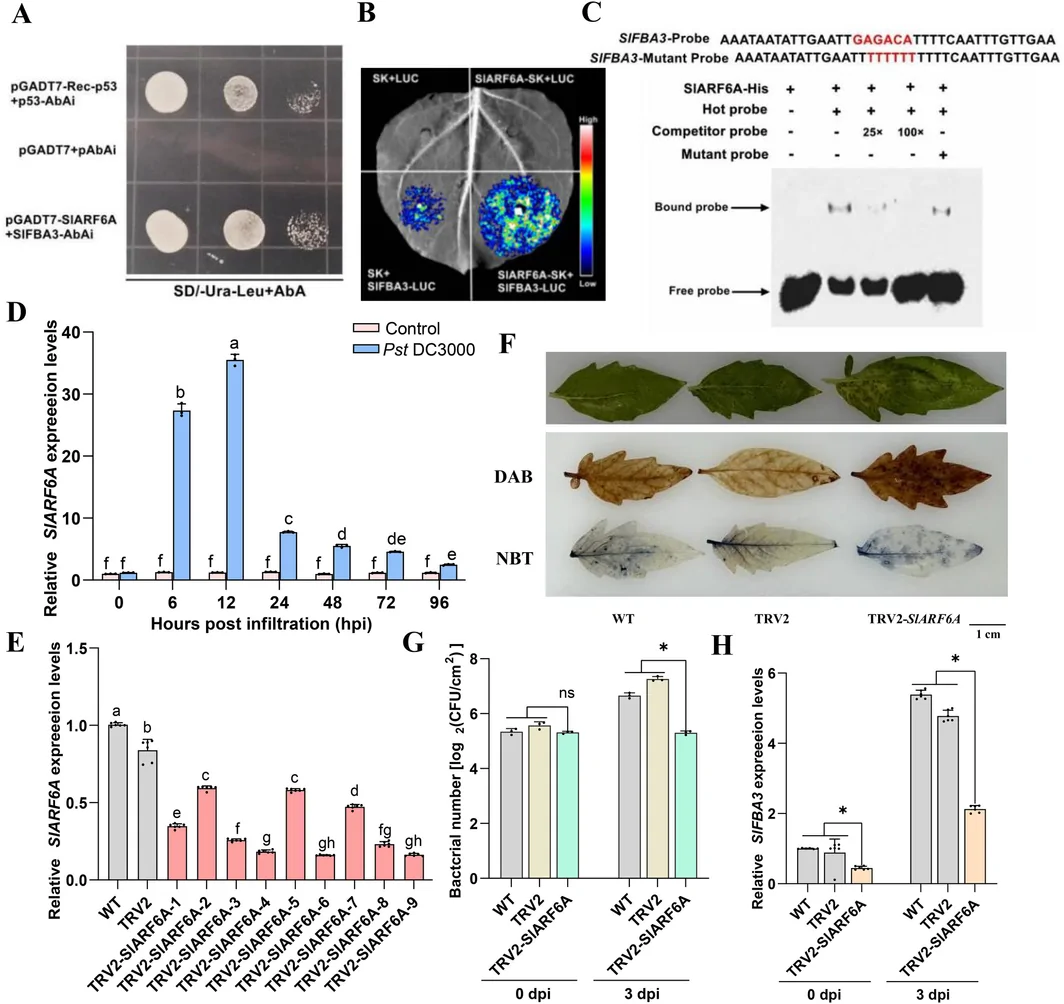
SlARF6A directly binds to the promoter of *SlFBA3* and positively regulates tomato resistance against *Pseudomonas syringae* pv. *tomato* (Pst) DC3000. (A) Yeast one‐hybrid (Y1H) analysis of the interaction between SlARF6A and the *SlFBA3* promoter. pGADT7‐*Rec‐p53*+*p53*‐AbAi and pGADT7+pAbAi were used as the positive and negative controls, respectively. (B) SlARF6A activated *SlFBA3* promoter luciferase (LUC) activity in *Nicotiana benthamiana*. (C) Electrophoretic mobility shift assay (EMSA) revealed that SlARF6A targets and regulates the ARF *cis*‐element in the promoter region of *SlFBA3*. The hot probes were biotin‐conjugated promoter fragments with specific ARF motifs, the competitive probe was an unlabelled probe, and the mutant probes were unlabelled fragments containing mutated nucleotides of the ARF motifs. (D) *SlARF6A* expression at different time points after Pst DC3000 was spray‐inoculated. One‐way ANOVA with Tukey's HSD; different letters indicate significant differences (*p* < 0.05). The error bars denote SDs (*n* = 6). (E) Virus‐induced gene silencing (VIGS) assay. The relative expression levels of *SlARF6A* in WT, TRV2 and TRV2‐*SlARF6A*. One‐way ANOVA with Tukey's HSD; different letters indicate significant differences (*p* < 0.05). The error bars denote SDs (*n* = 6). (F) Phenotypes, 3,3′‐diaminobenzidine (DAB) staining and nitroblue tetrazolium (NBT) staining of the WT, TRV2 and TRV2‐*SlARF6A* at 3 days post‐inoculation (dpi). (G) Bacterial counts on leaves of the WT, TRV2 and TRV2‐*SlARF6A* were measured at 0 and 3 dpi. (H) The relative expression levels of *SlFBA3* in WT, TRV2 and TRV2‐*SlARF6A* at 0 and 3 dpi. Asterisks indicate a significant difference (ns, not significant; *p < 0.05, **p < 0.01, Student's *t*‐test). The error bars denote SDs (*n* = 6).

To explore the upstream regulatory mechanism through which *SlFBA3* participates in disease resistance, we screened the transcription factors that could regulate *SlFBA3*. Through yeast one‐hybrid (Y1H) screening of a cDNA library using the *SlFBA3* promoter as bait, we discovered a transcription factor named SlARF6A. The Y1H assay results revealed that the yeast cells cotransfected with pGADT7‐*SlARF6A* and pAbAi‐*SlFBA3* grew on SD/−Ura−Leu+AbA selection medium, whereas the control cells did not grow (Figure 7A). The dual‐luciferase (LUC) results revealed that *SlARF6A*‐SK+*SlFBA3*‐LUC produced a significant LUC signal in *N. benthamiana*, suggesting that SlARF6A can target and regulate the expression of *SlFBA3* (Figure 7B). An electrophoretic mobility shift assay (EMSA) indicated that SlARF6A specifically recognized and bound to the ARF (TGTCTC) *cis*‐element (hot probe) in the *SlFBA3* promoter (Figure 7C). These results indicate that SlARF6A can specifically bind to and regulate the expression of the *SlFBA3* promoter both in vivo and in vitro.

We analysed the expression pattern of the transcription factor *SlARF6A* in the disease resistance response pathway and found that it significantly increased at 6 and 12 hpi (Figure 7D). To clarify the function of the *SlARF6A* gene in the disease resistance response, we generated *SlARF6A*‐silenced tomato plants using virus‐induced gene silencing (VIGS). Reverse transcription‐quantitative PCR (RT‐qPCR) analysis confirmed that compared with those in the WT and empty vector (TRV2) control plants, the *SlARF6A* transcript levels in the TRV2‐*SlARF6A* plants were significantly lower, indicating effective silencing (Figure 7E). After inoculation with Pst DC3000, the disease symptoms of the *SlARF6A*‐silenced plants were more severe with more lesions and significantly increased numbers of bacteria on the leaves compared with those of the WT and TRV2 plants (Figure 7F,G). Thus, silencing *SlARF6A* reduces the resistance of tomatoes to Pst DC3000. In addition, the expression level of *SlFBA3* in the TRV2‐*SlARF6A* plants was significantly lower than those in the WT and TRV2 plants, which further demonstrated that SlARF6A has a targeted regulatory effect on *SlFBA3* (Figure 7H). Therefore, *SlARF6A* acts as a positive regulator of disease resistance and directly targets the promoter of *SlFBA3* to activate its transcription.

## 3 Discussion

Fructose‐1,6‐bisphosphate aldolase (FBA), a key enzyme in the glycolytic pathway, has traditionally been regarded as mainly involved in stress responses through its regulation of carbon metabolism flow and energy supply within the plant body (Zhang et al. 2017). This study found that *SlFBA3* acts as a positive regulatory factor and plays a significant role in the defence response of tomatoes against Pst DC3000. However, its disease resistance function may not depend solely on the activity of metabolic enzymes. On the one hand, simple changes in metabolic flux are usually insufficient to achieve highly specific regulation of defence against pathogenic bacteria (Lacrampe et al. 2024; Hou et al. 2025). The rapid induction of *SlFBA3* expression in the early stage of infection and the significant disease resistance phenotype caused by its overexpression suggest that *SlFBA3* may participate in the transmission of disease resistance signals as a signalling molecule or regulatory hub. On the other hand, this study revealed that SlFBA3 can interact with a known negative regulatory factor, SlSGR, at the protein level. This physical interaction strongly indicates that SlFBA3 functions as a signalling molecule in the transmission and regulation of downstream defence responses. An increasing number of studies have shown that members of the plant FBA family of genes direct signal transduction functions in response to biotic and abiotic stresses, connecting metabolic status with the expression of downstream defence responses. For example, the biological function of ZmFBA8 in maize is not limited to catalysing metabolism but may also be involved in protein binding and signal transduction processes (Zhang et al. 2025). The functional loss mutants of soybean *GmFBAc1* and *GmFBAc2* not only show changes in carbon metabolism but also significantly affect the expression of genes related to the auxin (IAA) and JA signalling pathways (Qiu et al. 2023). These studies collectively support the function of FBA family proteins as signal regulatory factors in stress responses.

In plants, the balance between growth and defence is a central paradigm, where the activation of immune responses often occurs at the expense of developmental processes (Huot et al. 2014; Karasov et al. 2017). In this context, plants have evolved sophisticated regulatory mechanisms to fine‐tune this balance. The SGR family protein, known for its canonical function in chlorophyll degradation and senescence, has recently been discovered as a candidate player in this trade‐off (Sakuraba et al. 2012). In this study, we found that *SlSGR* also plays a negative regulatory role in the defence response of tomatoes against Pst DC3000. Overexpression of *SlSGR* compromised disease resistance, while its knockout significantly enhanced resistance. This negative regulation of the defence pathway helps plants prioritize growth under normal conditions, thereby maintaining the balance between growth and defence. Similar scenarios have been reported: In *Arabidopsis*, the transcription factor HB34 inhibits the JA signal to limit the immune response and thereby promotes the growth and development pathways (Li et al. 2025). Collectively, these findings underscore that negative regulation of defence pathways is a conserved strategy adopted by plants to prioritize growth under non‐stress conditions. Our discovery of *SlSGR* adds a new layer to this conserved paradigm and highlights the potential of manipulating such negative regulators for disease resistance breeding without compromising yield.

Notably, we found that SlFBA3 could partially antagonize the function of the negative regulatory factor SlSGR, thereby weakening the negative regulatory role of *SlSGR* in the disease response (Figure 6). Based on the protein interaction and functional antagonism observed between SlFBA3 and SlSGR, we speculate that there might be specific molecular mechanisms underlying this antagonism. Compared with the single *SlSGR*‐OE lines, *SlFBA3*‐OE/*SlSGR*‐OE dual‐overexpression lines had enhanced disease resistance. Therefore, we believe that SlFBA3 physically bound to SlSGR, preventing SlSGR from interacting with downstream target proteins (such as antioxidant regulatory proteins), thereby interfering with the negative regulatory function of SlSGR. Similar action patterns have been reported in plant immunity. For example, the effector protein CSEP0027 of *Blumeria graminis* f. sp. *hordei* directly binds to barley peroxidase HvCAT1, inhibiting the function of the peroxidase and disrupting the balance of ROS, thereby promoting the pathogenicity of the fungus (Yuan, Jin, et al. 2021). The effector AGLIP1 from *R. solani* interacts physically with OsAPX8, which inhibits the peroxidase activity of OsAPX8, thereby weakening the ROS clearance defence mechanism of the host and causing cell death (Fu et al. 2025). This study discovered the positive regulatory factor SlFBA3, which restores the antioxidant function by interacting with the negative regulatory factor SlSGR. This provides a new perspective for understanding how plants endogenously antagonize negative regulation. The specific manner in which SlFBA3 antagonizes SlSGR will be verified through further experiments.

**FIGURE 8:**
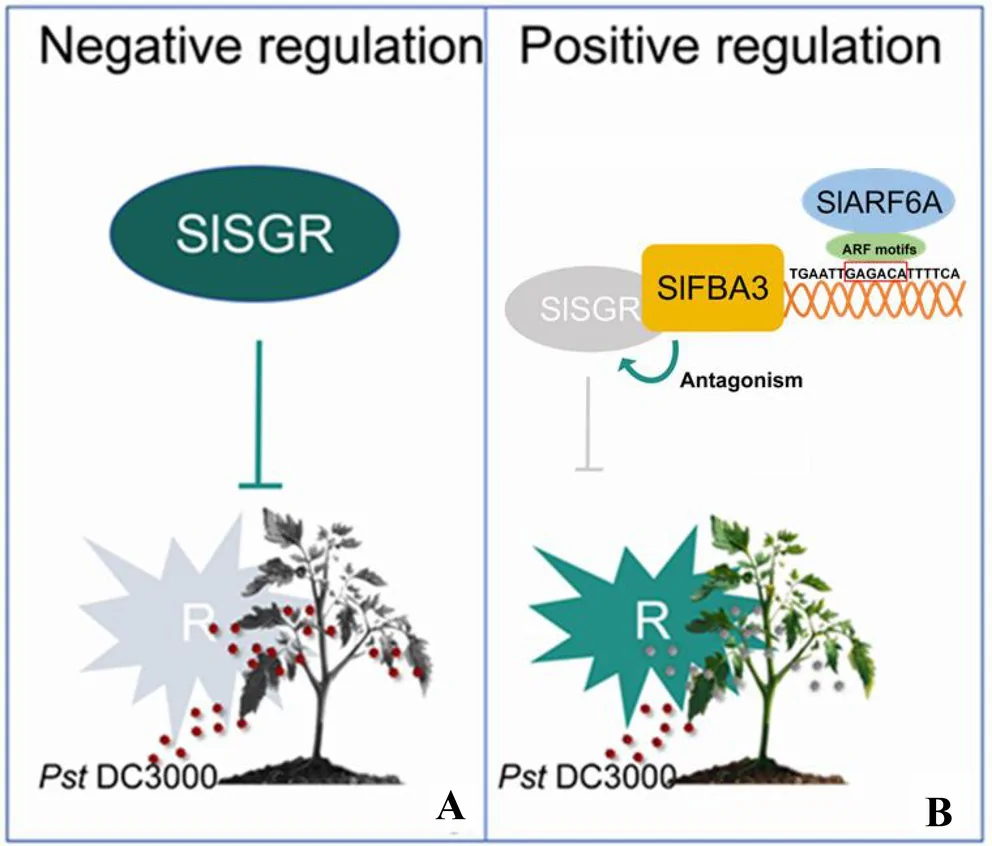
The SlARF6A–SlFBA3/SlSGR module in the defence response pathway of tomato against *Pseudomonas syringae* pv. *tomato* (Pst) DC3000. (A) The *SlSGR* gene negatively regulates Pst DC3000. (B) SlARF6A can regulate the expression of *SlFBA3* by targeting the ARF‐binding motif (GAGACA). Subsequently, the accumulated SlFBA3 protein interacts with the negative regulator SlSGR, thereby inhibiting the negative regulatory role of SlSGR in the disease response, and ultimately enhancing the resistance of tomato to Pst DC3000.

Auxin, an important plant hormone, not only regulates the growth and development of plants but also plays a significant role in the interaction between plants and pathogenic bacteria. Many pathogenic bacteria promote infection through the auxin signalling pathway (Kazan and Manners 2009). For example, mutation of the auxin response factor *SlARF8* can increase plant resistance to *B. cinerea* and *P. syringae* (Marash et al. 2024). The auxin response factors *ARF6* and *ARF8* are involved in regulating stomatal closure, thereby limiting pathogen invasion and enhancing resistance (Caruana et al. 2020). In this study, we observed that the auxin response factor SlARF6A can regulate the transcription of *SlFBA3* and positively regulate the defence response of tomato against Pst DC3000. This discovery provides new molecular evidence for the role of auxin signalling in plant immunity. Based on the above research, we constructed a SlARF6A–SlFBA3/SlSGR module to regulate disease resistance in tomato (Figure 8). This model links upstream transcriptional regulation, core functional proteins and downstream interacting factors in a complete signalling cascade, clearly demonstrating the molecular pathway involved in the progression from pathogen recognition to the establishment of disease resistance.

In conclusion, SlFBA3 and its interacting partner SlSGR play positive and negative regulatory roles, respectively, in the defence response of tomato against Pst DC3000. Under the induction of flg22 and csp22, *SlFBA3* can enhance the early immune signalling pathways, while *SlSGR* inhibits this response. Notably, SlFBA3 can partially antagonize the function of SlSGR, thereby weakening the negative regulatory role of SlSGR. In addition, *SlFBA3* can also be regulated by the positive regulatory factor SlARF6A. This study reveals that the SlARF6A–SlFBA3/SlSGR regulatory module plays an important role in the defence response of tomato.

## 4 Experimental Procedures

### 4.1 Plant Materials

The tomato variety 19,604 (WT) used in this experiment is a high‐generation inbred cultivar developed by the Tomato Molecular Genetics Breeding Laboratory of Northeast Agricultural University. The *SlFBA3*‐OE/*SlSGR*‐OE double overexpression lines were obtained by crossing *SlFBA3*‐OE and *SlSGR*‐OE lines. The cultivation conditions for the tomato plants were as follows: a day/night temperature of 25°C/18°C, 16 h light and 8 h darkness, and a relative humidity of 60%–70%.

### 4.2 Pathogen Infection and Physiological Index Measurement

Pst DC3000 was cultured on King's B agar supplemented with 50 mg L^−1^ rifampicin for 2 days at 28°C in the dark. Bacteria were collected and suspended in 10 mM MgCl_2_ solution. One‐month‐old plants were sprayed with a bacterial suspension (OD_600_ = 1) and cultivated for 3 days in an artificial climate chamber (25°C, 16 h light/8 h dark, 60% relative humidity).

Superoxide radical (O_2_^−^) was detected visually by the formation of dark blue precipitation after staining with 0.05% NBT solution. H_2_O_2_ was detected visually as a brown precipitate following DAB staining using 1 mg/mL DAB solution. NBT and DAB staining was performed according to the methods of Hu et al. (2019).

### 4.3 Bacterial Count Statistics

Leaves were randomly selected 3 days after inoculation, and samples were taken from the leaves using a 9 mm diameter punch. The sample was ground for 5 min using a sample grinder. 100 μL of the ground sample was diluted to 10^5^. 10 μL of the diluted solution was spread on King's B agar and incubated at 28°C, after which the number of Pst DC3000 bacterial colonies was analysed. CFU = [(Bacterial count × dilution factor × 50)/(6 × π × *r* ^2^)]. Three biological replicates were conducted for each sample.

### 4.4 RNA Extraction and RT‐qPCR Analysis

Total RNA was extracted from different plant tissues using a plant RNA extraction kit (TaKaRa Bio) according to the manufacturer's instructions. Reverse transcription of RNA into cDNA (1 μg of RNA from each sample) was performed using a HiScript III 1st Strand cDNA Synthesis Kit (+gDNA wiper) (Vazyme Biotech). The primers for *SlFBA3* (*Solyc02g084440*), *SlSGR* (*Solyc08g080090*), *SlFRK1* (*Solyc03g006860*), *SlPR1* (*Solyc09g007010*), *SlWRKY22* (*Solyc01g095100*) and *SlNAC* (*Solyc02g069960*) genes are shown in Table S1. Tomato elongation factor‐1a (*SlEF‐1a*) was used as an internal standard. All the gene‐specific primers used for RT‐qPCR in this study are listed in Table S1. The relative expression was quantified and calculated using the 2^−ΔΔ*C*t^ method. Three biological replicates were conducted for each sample.

### 4.5 Subcellular Localization

The coding sequences (CDSs) of *SlFBA3* and *SlSGR* were amplified (the primers are shown in Table S1) and cloned and inserted into pCAMBIA2300‐EGFP using the CloneExpress II One Step Cloning Kit (Vazyme). *SlFBA3* and *SlSGR* formed fusion proteins with the EGFP tag. *N. benthamiana* leaves were infiltrated with the constructs carried by *Agrobacterium tumefaciens* GV3101. After approximately 48 h, the fluorescence signal was detected using an FV3000 confocal microscope (TCS SP8; Olympus).

### 4.6 Plant Transformation

Two single‐guide RNAs (sgRNAs) of *SlFBA3* and *SlSGR* were designed using the CRISPR tool (http://cbi.hzau.edu.cn/cgi‐bin/CRISPR) (Lei et al. 2014). Two sgRNAs of each gene were subsequently cloned and inserted into pYL‐CRISPR/Cas9 vector. The sequences of the sgRNAs are shown in Table S1. The first‐generation transformed plants (T_0_) were genotyped using forward and reverse primers flanking the sgRNA target sites (Table S1). The full‐length CDSs of *SlFBA3* and *SlSGR* without a terminator were cloned and inserted into the pCAMBIA1300‐HA vector and expressed as *SlFBA3*‐HA and *SlSGR*‐HA fusion proteins. The vector was digested using SalI and KpnI restriction endonuclease enzymes. The primers used for gene amplification and transformed plant detection are shown in Table S1. The binary vectors were subsequently transformed into the WT by *Agrobacterium*‐mediated genetic transformation according to the protocol of Van Eck et al. (2019).

### 4.7 H_2_O_2_ Content and CAT Activity

The content of H_2_O_2_ was determined, 0.5 g of sample was extracted with 5 mL phosphate‐buffered saline (PBS), and ground into homogenate at 4°C, centrifuged at 12,000 *g* for 10 min, and repeated. The supernatant was collected for H_2_O_2_ content analysis using a kit according to the manufacturer's recommendations (A064‐1, Nanjing). The content of CAT was determined, 0.1 g of sample was extracted with 1 mL PBS, and ground into homogenate at 4°C, centrifuged at 12,000 *g* for 10 min, and repeated. The supernatant was collected for CAT activity analysis using a kit according to the manufacturer's recommendations (G0105F, Suzhou).

### 4.8 flg22 and csp22 Peptide Treatments

The leaves of tomato at the 4‐leaf stage were treated with 1 μM flg22 (PhytoTechnology Laboratories) or 1 μM csp22 (Leuschen‐Kohl et al. 2025). The csp22 peptide sequence is ATGTVKWFNETKGFGFITPDGG (Wei et al. 2018), with a purity of ≥ 95% purity (Sangon Biotech). The tomato seedlings were sampled for testing 0, 6 and 12 h after being treated with flg22 and csp22. Sterile distilled water was used for untreated controls.

### 4.9 ROS Burst Assay

The ROS burst assay was performed following the protocol of Yuan, Jiang, et al. (2021) with minor adjustments. Leaf slices (0.2 cm^2^) were randomly selected and placed in the wells of a 96‐well plate. 100 μL of double‐distilled water was added to each well and the plates incubated in an incubator at a constant temperature. After incubation, 200 μL of chemiluminescent mix buffer was added to each well. The buffer contained 100 μg/mL peroxidase (Coolaber), 200 μM luminol (Coolaber), and 200 nM flg22 (or 100 nM csp22). The chemiluminescence signal was recorded using the SpectraMax iD3 microplate luminometer (Beckman Coulter) for 40 min. Each treatment included six leaves from different tomatoes.

### 4.10 MAPK Induction Assay

The tomato seedlings aged 4–5 weeks were treated with 1 μM flg22, 1 μM csp22 or water for 15 min, and then frozen with liquid nitrogen. The activation of MAPK was detected using phosphorylation‐specific antibodies by western blotting. The phospho‐p44/42 MAPK (Erk1/2) (Thr202/Tyr204) antibody (Cell Signalling, 4730, 1:2000 dilution) was diluted according to the manufacturer's instructions. The imaging was performed using ECL chemiluminescence method. The total protein was verified by anti‐Actin (Abmart, M20009, 1:5000 dilution) for equal sample loading.

### 4.11 Y2H Assay

The CDSs of *SlFBA3* were cloned and inserted into the pBT3‐N vector, and *SlSGR* was cloned and inserted into a pPR3‐N vector. The pPR3‐N‐*SlSGR* and pBT3‐N‐*SlFBA3* were co‐transfected into the yeast line NMY51. Yeast transformants were subsequently identified by culture on SD/−Leu−Trp (SD/−L−T, double dropout) and SD/−Leu−Trp−His−Ade (SD/−L−T−H−A, quadruple dropout) media (Clontech). The primers used for vector construction and amplification of the coding sequences of the prey proteins are shown in Table S1.

### 4.12 BiFC and LCI Assays

To generate constructs for the BiFC assays, the CDSs of *SlFBA3* were inserted into pSPYNE to generate *SlFBA3*‐NE. The CDSs of *SlSGR* were cloned and inserted into pSPYCE vectors to generate *SlSGR*‐CE. The primers used for vector construction are shown in Table S1. *N. benthamiana* leaves were infiltrated with the constructs (*SlFBA*3‐NE + *SlSGR*‐CE) carried by *A. tumefaciens* GV3101. After approximately 48 h, the fluorescence signals were detected using an FV3000 confocal microscope (TCS SP8; Olympus).

To produce constructs for the LCI assay, the CDSs of *SlFBA3* were inserted into the pCAMBIA1300‐Nluc vector. The CDSs of *SlSGR* were inserted into the pCAMBIA1300‐Cluc vector. The primers used for vector construction are shown in Table S1. The constructed vectors were transferred into *A. tumefaciens* GV3101, which was subsequently infiltrated into *N. benthamiana* leaves. Two days later, the leaves were sprayed with 1 mM luciferin solution to determine the luminescence intensity. After 10 min of incubation in the dark, the interaction intensity was determined using a luminescence imaging system (GelView 6000Plus, Biolight).

### 4.13 Co‐IP Assay

*SlSGR*‐EGFP and *SlFBA*3‐HA were constructed with the primers listed in Table S1. The *SlSGR*‐EGFP and *SlFBA3*‐HA *Agrobacterium* strains were coinfiltrated into *N. benthamiana* leaves. Approximately 48 h later, the leaves were collected for Co‐IP according to methods described previously (Wang et al. 2021). The total protein was extracted from the *N. benthamiana* leaves using lysis buffer supplemented with a protease inhibitor cocktail. Total protein from each sample was incubated with anti‐GFP magnetic beads (BeyoMag Anti‐GFP Magnetic Beads, Beyotime) for 4 h at 4°C. The magnetic beads were then washed three times with 1 mL of lysis buffer on a magnetic stand. To detect the HA‐tagged proteins that were immunoprecipitated with the EGFP‐tagged proteins, immunoblotting was performed using anti‐GFP (Abmart, M20004, used at 1:2000 dilution) and anti‐HA antibodies (Sigma‐Aldrich, H3663, 1:1000 dilution).

### 4.14 Transient Expression Assays

For transient expression assays, the leaves of WT and *SlFBA3*‐OE plants were inoculated with *A. tumefaciens* GV3101 carrying p35S:*SlSGR*‐EGFP and p35S‐EGFP (as the control) at an OD_600_ value of 1.0. At 72 hpi, the expression of *SlSGR* was confirmed by RT‐qPCR. Subsequently, the plants were inoculated with Pst DC3000 at a concentration of 1 × 10^7^ CFU/mL. The disease symptoms were evaluated 3 days post‐inoculation (dpi). The experiment was conducted with three independent biological replicates.

### 4.15 Y1H Assay

To investigate the interaction between the transcription factor *SlARF6A* and the promoter of *SlFBA3*, we conducted a Y1H assay experiment. The full‐length CDS of *SlARF6A* was amplified with specific primers (Table S1) and inserted into the pGADT7 vector. A fragment of the *SlFBA3* promoter region was amplified and inserted into the pAbAi vector. The recombinant pAbAi‐*SlFBA3* plasmid was linearized and transformed into the Y1HGold yeast strain. The prey plasmid *SlARF6A*‐AD was transformed into Y1HGold recipient cells harbouring the pAbAi‐*SlFBA3* bait. The transformed yeast cells were subsequently plated onto SD/−Leu/−Ura medium supplemented with aureobasidin A (AbA) and incubated at 30°C for 3–5 days.

### 4.16 Dual‐Luciferase (Dual‐LUC) Activity Assays

*Cis*‐acting regulatory elements in the promoter sequence of *SlFBA3* were predicted using the PlantCARE database (http://bioinformatics.psb.ugent.be/webtools/plantcare/html/). The full‐length CDS of *SlARF6A* was amplified and inserted into the pCaMV35S‐SK vector (the effector construct). The promoter region of *SlFBA3* was amplified and inserted into the pCaMV35S‐LUC vector (the reporter construct). The primers used for these manipulations are listed in Table S1. *SlARF6A*‐SK and *SlFBA3*‐LUC were transferred into *A. tumefaciens* GV3101 (pSoup+p19) and adjusted to an OD_600_ of 0.3. Equal volumes of cultures containing the effector and reporter constructs were mixed and coinfiltrated into the abaxial side of *N. benthamiana* leaves using a needleless syringe. The infiltrated plants were first incubated in the dark at 21°C for 1 day, followed by 2 days under a 16‐h light/8‐h dark photoperiod at the same temperature. To detect luciferase activity, the leaves were sprayed with 300 μg/mL D‐luciferin potassium salt and placed in the dark for 10 min. Luminescence signals were then captured using a CCD imaging system (Tanon‐5200).

### 4.17 EMSA

The full‐length CDS of *SlARF6A* was inserted into the pET‐28a vector to generate the *SlARF6A*‐His fusion construct, which was subsequently transformed into *Escherichia coli* BL21 (DE3) competent cells. Recombinant protein expression was induced with 1 mM IPTG, and the protein was purified using a His‐tag protein purification kit (Beyotime). For the binding assay, three types of double‐stranded DNA probes were synthesized (primers listed in Table S1): a biotin‐labelled probe containing the ARF *cis*‐element derived from the *SlFBA3* promoter (hot probe), an unlabelled competitor probe with the same sequence (cold probe), and a biotin‐labelled mutant probe with nucleotide substitutions in the ARF core sequence. An EMSA was performed using a LightShift Chemiluminescent EMSA kit (Thermo Scientific). Purified *SlARF6A*‐His protein was incubated with the probes according to the manufacturer's protocol. Protein–DNA complexes were resolved on a 4% native polyacrylamide gel and visualized using a chemiluminescence imaging system.

### 4.18 VIGS Assay

For the VIGS assay, specific primers targeting *SlARF6A* were designed using the VIGS tool available on the Sol Genomics Network (https://solgenomics.net/) to amplify a gene‐specific fragment (primer sequences are listed in Table S1). The amplified product was inserted into the pTRV2 vector. The resulting construct (pTRV2‐*SlARF6A*) and the pTRV2 control were transformed into *A. tumefaciens* GV3101. *Agrobacterium* cultures carrying pTRV1 were mixed with those containing either pTRV2 or pTRV2‐*SlARF6A* at a 1:1 ratio (OD_600_ = 0.3). The tomato plants were then inoculated using a syringe. The silencing efficiency was evaluated by RT‐qPCR after 21 days.

## Author Contributions

**Tong Pei:** conceptualization, investigation, data curation, writing – original draft. **Weiman Sun:** investigation, methodology, validation. **Tairu Wu:** software, validation, investigation. **Dalong Li:** investigation. **He Zhang:** investigation. **Xiangyang Xu:** funding acquisition, supervision. **Tingting Zhao:** conceptualization, writing – review and editing, supervision.

## Funding

This study was supported by the Regional Innovation and Development Joint Fund of the National Natural Science Foundation of China (U22A20496), Funding for expert positions in the national vegetable industry technology system (CARS‐23‐A11), Key research and development plan of Heilongjiang Province (SC2022ZX02C0202).

## Conflicts of Interest

The authors declare no conflicts of interest.

## Supporting information


**Figure S1:** Identification of *SlFBA3* transgenic tomato plants.

**Figure S2:** Split‐ubiquitin yeast two‐hybrid (Y2H) system suitability verification and autoactivation verification.

**Figure S3:** Identification of *SlSGR* transgenic tomato plants.

**Figure S4:** The resistance test of the *SIFBA3/SlSGR* module against *Xanthomonas euvesicatoria* .

**Figure S5:** Western blot was used to detect the accumulation levels of SlFBA3 and SlSGR proteins in the *SlFBA3*‐OE/*SlSGR*‐OE double overexpression lines.

**Table S1:** Primers used in this study.

## Data Availability

The data supporting the findings of this study are available in the main text and Supporting Information.
