# Supplementary material for: The SlARF6A‐SlFBA3/SlSGR Module Regulates the Resistance of Tomatoes to Pseudomonas syringae pv. tomato
Source: Mol Plant Pathol. 2026 Aug 21;27(8):e70327. doi: 10.1111/mpp.70327 (PMC13498865; doi:10.1111/mpp.70327)
Supplement: Supplementary file 1 [file MPP-27-e70327-s001.docx]

**FIGURE S5** Western blot was used to detect the accumulation levels of SlFBA3 and SlSGR proteins in the *SlFBA3*-OE/*SlSGR*-OE double overexpression lines. Protein immunoblotting using HA antibody was performed on four overexpression lines.
