# Supplementary material for: The SlARF6A‐SlFBA3/SlSGR Module Regulates the Resistance of Tomatoes to Pseudomonas syringae pv. tomato
Source: Mol Plant Pathol. 2026 Aug 21;27(8):e70327. doi: 10.1111/mpp.70327 (PMC13498865; doi:10.1111/mpp.70327)
Supplement: Supplementary file 2 [file MPP-27-e70327-s002.docx]

100bp

250bp

500bp

OE-1

OE-3

OE-2

OE-4

OE-5

WT

OE-6

OE-7

OE-8

OE-9

OE-10

**A**

**B**

OE-3

OE-6

OE-9

OE-10

**FIGURE S1** Identification of *SlFBA*3 transgenic tomato plants. (A) PCR identification of positive plants in the *SlFBA3* overexpression lines. (B) Western blot was used to detect the protein accumulation level of SlFBA3 in the *SlFBA*3-OE strain. Protein immunoblotting using HA antibody was performed on four overexpression lines (OE).
