# Supplementary material for: The SlARF6A‐SlFBA3/SlSGR Module Regulates the Resistance of Tomatoes to Pseudomonas syringae pv. tomato
Source: Mol Plant Pathol. 2026 Aug 21;27(8):e70327. doi: 10.1111/mpp.70327 (PMC13498865; doi:10.1111/mpp.70327)
Supplement: Supplementary file 3 [file MPP-27-e70327-s003.docx]

**FIGURE S4** The resistance test of the *SIFBA3*-*SlSGR* module against *Xanthomonas euvesicatoria*. (A) *SlFBA3* expression at different time points after the *Xanthomonas euvesicatoria* was sprayed. (B) *SlSGR* expression at different time points after the *Xanthomonas euvesicatoria* was sprayed. One-way ANOVA with Tukey's HSD; different letters indicate significant differences (p < 0.05). The error bars denote SDs (n =3). (C) Phenotypes of the WT, *SlFBA3*-OE, *SlFBA3*-CR, *SlSGR*-OE and *SlSGR*-CR at 3 dpi (10^8^ CFU/mL). Upper panel: spray inoculation; lower panel: syringe infiltration. (D) H_2_O_2_ content and CAT activity of the WT, *SlFBA3*-OE, *SlFBA3*-CR, *SlSGR*-OE and *SlSGR*-CR at 0, 12 and 72 hpi. (E) The expression levels of the disease-resistant gene *SlFRK1*, *SlPR1*, *SlWRKY22* and *SlNAC* in WT, *SlFBA3*-OE, *SlFBA3*-CR, *SlSGR*-OE and *SlSGR*-CR lines at 0, 12 and 72 hpi. Asterisks indicate a significant difference (ns, no significant; *P<0.05; **P<0.01, Student’s t-test). The error bars denote SDs (n=6).

**E**

**C**

**D**

**A**

**B**

***slfba3-*CR**

**WT**

***SlFBA3*-OE**

***SlSGR-*OE**

***slsgr-*CR**

**1 cm**
