# Supplementary material for: The SlARF6A‐SlFBA3/SlSGR Module Regulates the Resistance of Tomatoes to Pseudomonas syringae pv. tomato
Source: Mol Plant Pathol. 2026 Aug 21;27(8):e70327. doi: 10.1111/mpp.70327 (PMC13498865; doi:10.1111/mpp.70327)
Supplement: Supplementary file 4 [file MPP-27-e70327-s004.docx]

**FIGURE S2** Split-ubiquitin Y2H system suitability verification and autoactivation verification. Yeast carrying SlFBA3-pBT3-N + pOst1-NubI grew on selective medium demonstrates that split-ubiquitin Y2H system is suitable for *SlFBA3*; Yeast carrying SlFBA3-pBT3-N + pPR3-N didn’t grow on selective medium demonstrates that there is no autoactiviation for *SlFBA3*; pTSU2-APP + pNubG-Fe65 is the positive control; pTSU2-APP + pPR3-N is the negative control.
